# Cytosolic Quality Control of Mislocalized Proteins Requires RNF126 Recruitment to Bag6

**DOI:** 10.1016/j.molcel.2014.05.025

**Published:** 2014-07-17

**Authors:** Monica C. Rodrigo-Brenni, Erik Gutierrez, Ramanujan S. Hegde

**Affiliations:** 1 MRC Laboratory of Molecular Biology, Francis Crick Avenue, Cambridge CB2 0QH, UK; 2 Laboratory of Gene Regulation and Development, *Eunice Kennedy Shriver* National Institute of Child Health and Human Development, National Institutes of Health, Bethesda, MD 20892, USA

## Abstract

Approximately 30% of eukaryotic proteins contain hydrophobic signals for localization to the secretory pathway. These proteins can be mislocalized in the cytosol due to mutations in their targeting signals, certain stresses, or intrinsic inefficiencies in their translocation. Mislocalized proteins (MLPs) are protected from aggregation by the Bag6 complex and degraded by a poorly characterized proteasome-dependent pathway. Here, we identify the ubiquitin ligase RNF126 as a key component of the MLP degradation pathway. In vitro reconstitution and fractionation studies reveal that RNF126 is the primary Bag6-dependent ligase. RNF126 is recruited to the N-terminal Ubl domain of Bag6 and preferentially ubiquitinates juxtahydrophobic lysine residues on Bag6-associated clients. Interfering with RNF126 recruitment in vitro prevents ubiquitination, and RNF126 depletion in cells partially stabilizes a Bag6 client. Bag6-dependent ubiquitination can be recapitulated with purified components, paving the way for mechanistic analyses of downstream steps in this cytosolic quality control pathway.

## Highlights

• The chaperone Bag6 recruits the ubiquitin ligase RNF126 to its Ubl domain
• RNF126 is necessary and sufficient for optimal ubiquitination of Bag6 clients
• Purified Bag6-client complex supports ubiquitination by recombinant RNF126
• Bag6-associated mislocalized protein is stabilized by loss of RNF126 in cells

## Graphical Abstract

**Figure:**
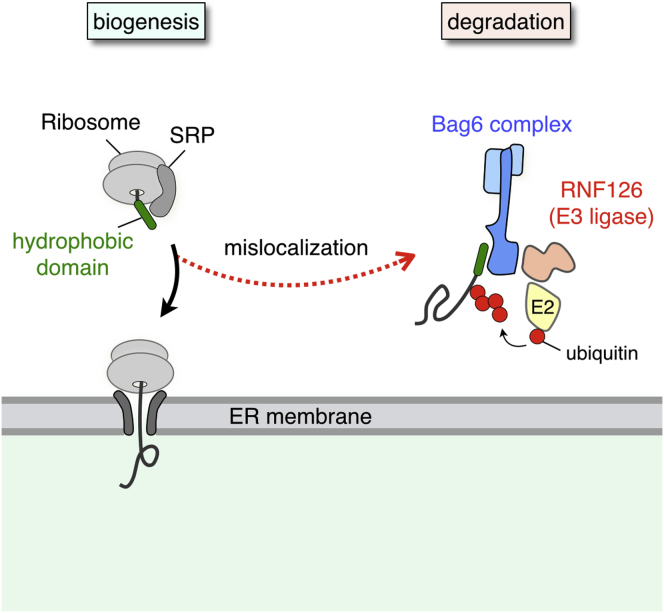

## Teaser

Proteins that fail to be localized to the correct compartment must be degraded to avoid the cellular damage caused by their accumulation. These mislocalized proteins are recognized by the Bag6 complex. The study by Rodrigo-Brenni et al. finds that Bag6 recruits a ubiquitin ligase called RNF126 that tags mislocalized proteins for degradation.

## Introduction

Protein quality control is essential for cellular homeostasis (Kim et al., 2013). Failure to promptly recognize and degrade defective or superfluous proteins leads to their accumulation. This accumulation of aberrant proteins can have several consequences, including protein aggregation, inappropriate interactions, damage to cellular membranes, induction of stress responses, and many others. Each of these consequences can be detrimental at both the cellular and organismal level, and is causative of numerous human diseases (Cuanalo-Contreras et al., 2013). Thus, deciphering the molecular basis of protein misfolding diseases requires an understanding of the cell’s various protein quality control pathways.

Proteins in most, if not all, cellular compartments are subjected to quality control. The cytosol is the largest cellular compartment and houses the most diverse proteome. The range of proteins it handles, and the myriad ways in which they can be defective, requires a highly flexible quality control system. This challenging task is not handled by a single pathway, but by multiple parallel pathways dedicated to different types of aberrant proteins (Buchberger et al., 2010; Fang and Mayor, 2012; Rodrigo-Brenni and Hegde, 2012). The pathways range widely, including ribosome-associated systems for partially synthesized proteins (Pechmann et al., 2013), chaperone-assisted pathways of degradation (Kettern et al., 2010), and autophagy-based pathways for large multiprotein aggregates (Jimenez-Sanchez et al., 2012). A current goal in the quality control field is to define the full complement of pathways, the components that comprise each pathway, and their respective client specificities.

Earlier work has shown that one cellular process requiring quality control is protein translocation into organelles. For example, import into the endoplasmic reticulum is not perfectly efficient, resulting inevitably in at least some polypeptides mislocalized to the cytosol (Levine et al., 2005; Rane et al., 2004). This mislocalization can be enhanced during ER stress (Kang et al., 2006; Orsi et al., 2006), by rare mutations in signal peptides (Arnold et al., 1990; Hussain et al., 2013), and possibly by mutant translocation machinery (Zimmermann et al., 2006). Importantly, protein mislocalization can lead to disease in both animal models and humans (Arnold et al., 1990; Mesbah et al., 2006; Rane et al., 2004; Zimmermann et al., 2006). Thus, cells are likely to have evolved mechanisms to deal with mislocalized proteins (MLPs) to avoid their accumulation.

Previous studies have shown that ∼10%–20% of mammalian prion protein (PrP) is mislocalized to the cytosol, and this population of PrP is rapidly degraded via the ubiquitin-proteasome system (Drisaldi et al., 2003; Rane et al., 2004). Using mislocalized PrP as a model substrate, an in vitro system was used to search for protein factors involved in its ubiquitination (Hessa et al., 2011). A combination of crosslinking and functional analyses led to the identification of the heterotrimeric Bag6 complex as a factor that interacts specifically with the unprocessed hydrophobic domains of PrP and other MLPs (Hessa et al., 2011). Bag6 complex was necessary for maximal MLP ubiquitination in vitro, and for efficient degradation of mislocalized PrP in cultured cells. Thus, the Bag6 complex was proposed to be a component of the quality control pathway for MLPs.

Parallel studies widened the scope of Bag6 in protein quality control. In one set of studies, Bag6 was found to interact with proteins dislocated from the endoplasmic reticulum (Claessen and Ploegh, 2011; Wang et al., 2011). In this case, Bag6 appears to interact after substrate ubiquitination, and is needed to maintain client solubility until delivery to the proteasome. Other studies found Bag6 associated with newly synthesized polyubiquitinated proteins that were proposed to generate peptides for MHC class I presentation (Minami et al., 2010). Collectively, these findings implicate Bag6 in multiple quality control pathways (Kawahara et al., 2013), although its exact role in any of them remains poorly understood.

The heterotrimeric Bag6 complex is composed of Bag6, TRC35, and Ubl4A (Mariappan et al., 2010). In addition to mediating ubiquitination, the Bag6 complex was shown to be involved in the capture and loading of tail-anchored (TA) membrane proteins onto the targeting factor TRC40 (Mariappan et al., 2010). The TRC35 and Ubl4A subunits appear to be conserved in all eukaryotes and participate in the TA targeting pathway (Wang et al., 2010). By contrast, the Bag6 subunit appears to be a later evolutionary acquisition that has embellished a targeting factor complex with protein quality control capability. It was therefore proposed that the Bag6 complex is at the center of a triage reaction that routes hydrophobic proteins toward either targeting (in the case of TA proteins) or degradation (for other hydrophobic proteins) (Hessa et al., 2011).

Photocrosslinking and mutagenesis experiments demonstrated that Bag6 interacts with hydrophobic domains (Hessa et al., 2011; Leznicki et al., 2010; Mariappan et al., 2010). The client then becomes ubiquitinated in a reaction that does not seem to require TRC35 or Ubl4A, but does need the ubiquitin-like (Ubl) domain at the N terminus of Bag6 (Hessa et al., 2011). These observations led to a model in which the Ubl domain recruits a ubiquitin ligase to target Bag6-associated proteins for degradation. However, alternative explanations, such as Bag6 itself acting as an atypical ligase, could not be excluded. Thus, the mechanism of ubiquitination in the MLP degradation pathway was unknown. Here, we have continued our investigation of MLP degradation and identify RNF126 as a Bag6-dependent ubiquitin ligase in this pathway.

## Results

### Characterization of a Bag6-Dependent E3 Ligase Activity

We first established whether, as suggested indirectly from earlier work (Hessa et al., 2011), a factor(s) in addition to the Bag6 complex is required for ubiquitination of MLPs. Our strategy was to use fractionation of a crude in vitro MLP ubiquitination system to find a situation in which ubiquitination activity was lost despite containing all known factors of this pathway. Our model MLP was an artificial protein (termed TR-β) containing two transmembrane domains (TMDs) that serve as Bag6 recruitment sites (Hessa et al., 2011). Mislocalization of TR-β was enforced by its in vitro translation in reticulocyte lysate lacking ER microsomes. The reaction contained ^35^S-methionine to selectively radiolabel the newly synthesized MLP, and His-tagged ubiquitin to facilitate downstream purification of ubiquitinated products as needed.

**Figure 1:**
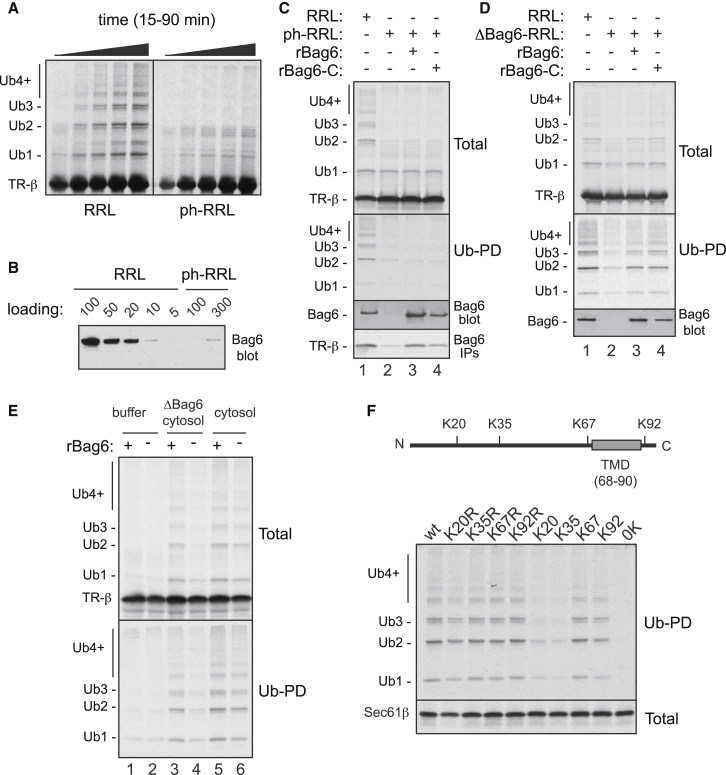
Characterization of a Bag6-Dependent Ligase for Mislocalized Proteins (A) ^35^S-methionine-labeled TR-β was in vitro translated for various times using rabbit reticulocyte lysate (RRL, left) or phenyl-depleted RRL (ph-RRL, right) and analyzed by SDS-PAGE and autoradiography. Unmodified TR-β and its ubiquitinated species are indicated. (B) Levels of Bag6 in different relative amounts of RRL and ph-RRL were determined by immunoblot. (C) Radiolabeled TR-β was produced in RRL, ph-RRL, or ph-RRL supplemented with recombinant Bag6 (rBag6) or Bag6 complex (rBag6-C). All reactions contained His-tagged ubiquitin. The translation products were analyzed directly (Total) or after His-ubiquitin pull-downs (Ub-PD). The level of Bag6 in each reaction was measured by immunoblotting (Bag6 blot). Bag6 interaction with TR-β was assessed by visualizing the amount of radiolabeled TR-β in anti-Bag6 immunoprecipitations (Bag6 IPs). (D) TR-β was translated in control (RRL) or Bag6-depleted (ΔBag6-RRL) lysates without or with readdition of rBag6 or rBag6-C as indicated. Reactions were analyzed directly by SDS-PAGE and autoradiography (Total) or after ubiquitin pull-downs (Ub-PD). Bag6 in each reaction was analyzed by immunoblot. (E) Radiolabeled TR-β translated for 30 min in ph-RRL in the presence (+) or absence (−) of rBag6 was subsequently incubated with buffer, total RRL (cytosol), or Bag6-depleted RRL (ΔBag6 cytosol). TR-β ubiquitination was followed by SDS-PAGE and autoradiography directly (Total) or after ubiquitin pull-downs (Ub-PD). (F) Diagram depicting location of lysines and transmembrane domain (TMD) in Sec61β. Wild-type Sec61β, lysine-to-arginine mutants (KxR, where x denotes residue number) or constructs containing a single lysine (Kx, where x denotes residue number of the single lysine) were in vitro translated and analyzed directly (Total) or after ubiquitin pull-downs (Ub-PD). See also Figure S1.

We found that while the complete translation system supports MLP ubiquitination, a translation extract passed over phenyl-Sepharose was diminished in MLP ubiquitination (Figure 1A). Another ubiquitination pathway that uses the same E1 and E2 enzymes as the Bag6 pathway was not affected by phenyl-Sepharose depletion (Shao et al., 2013), indicating that these components are not responsible for the MLP ubiquitination defect. By contrast, Bag6 complex was depleted by more than 95% (Figure 1B), suggesting a potentially simple explanation for diminished MLP ubiquitination. However, neither recombinant Bag6 (rBag6; Figure S1A available online) nor the heterotrimeric Bag6 complex (rBag6-complex; Figure S1B) could substantially improve the ubiquitination deficiency in phenyl-depleted lysate (Figure 1C).

Two observations verified that rBag6 and rBag6-complex are functional. First, both rBag6 and rBag6-complex could interact with MLPs in the phenyl-depleted lysate as judged by coimmunoprecipitation (Figure 1C) and crosslinking (data not shown). Second, both factors could restore ubiquitination to IVT lysate immunodepleted of Bag6 complex (Figure 1D). This suggested that phenyl-Sepharose depletes at least one factor in addition to Bag6 that is required for efficient MLP ubiquitination. We posited that the missing factor may be a ubiquitin ligase (E3) needed for maximal MLP ubiquitination. If this putative MLP ligase acts in the same pathway as Bag6, then its action should be Bag6 dependent.

To examine this idea, an MLP was translated in phenyl-depleted lysate (i.e., lacking both Bag6 and the presumptive ligase) supplemented with either nothing or rBag6. The MLP produced under these conditions was not effectively ubiquitinated. These reactions were then mixed with either buffer, complete cytosol, or Bag6-immunodepleted cytosol and incubated further before evaluation for ubiquitination. All reactions contained excess E1, E2, ubiquitin, and ATP to ensure these components were not limiting. Minimal ubiquitination was observed without cytosol (Figure 1E, lanes 1 and 2), while complete cytosol mediated ubiquitination of the MLP, regardless of whether it was produced in the absence or presence of rBag6 (Figure 1E, lanes 5 and 6). By contrast, cytosol immunodepleted of Bag6 complex could only restore ubiquitination to MLP produced in the presence of rBag6 (Figure 1E, lanes 3 and 4). This suggested that Bag6-depleted cytosol contains a ligase for MLP ubiquitination that only operates in the presence of Bag6.

Since the putative MLP ligase is Bag6 dependent, we wondered if it exhibits preference for client lysines near the site(s) of Bag6 recognition. For ease of interpretation, we analyzed the TA protein Sec61β as a client, as it contains four evenly spaced lysines and a single TMD that binds Bag6 (Figure 1F, diagram). Versions of Sec61β containing only K67 or K92 (flanking the TMD) were ubiquitinated as well as native Sec61β (Figure 1F). By contrast, Sec61β constructs containing only K20 or only K35 were sharply reduced in their ubiquitination. Similar results were seen with an MLP containing two TMDs in which most lysines near the TMDs were preferred targets for ubiquitination (Figure S1C). These observations suggest that lysines immediately adjacent to Bag6 binding sites (i.e., TMDs) are favored by the ubiquitination machinery, consistent with the idea that the ligase accesses MLPs via Bag6.

### A Purified Bag6-Client Complex Supports Ubiquitination

While the Bag6-dependent pathway is the primary source of MLP ubiquitination in vitro, it was clear from the Bag6 and phenyl depletion experiments that alternative (albeit less efficient) ubiquitination pathways operate in the absence of Bag6. This existence of multiple pathways limited the overall signal-to-noise of Bag6 pathway analysis in this system, prompting us to develop a more refined assay. Because we could experimentally uncouple MLP loading onto Bag6 from its subsequent ubiquitination, we reasoned that a purified Bag6-client complex would be the ideal starting point for studying this pathway in isolation.

**Figure 2:**
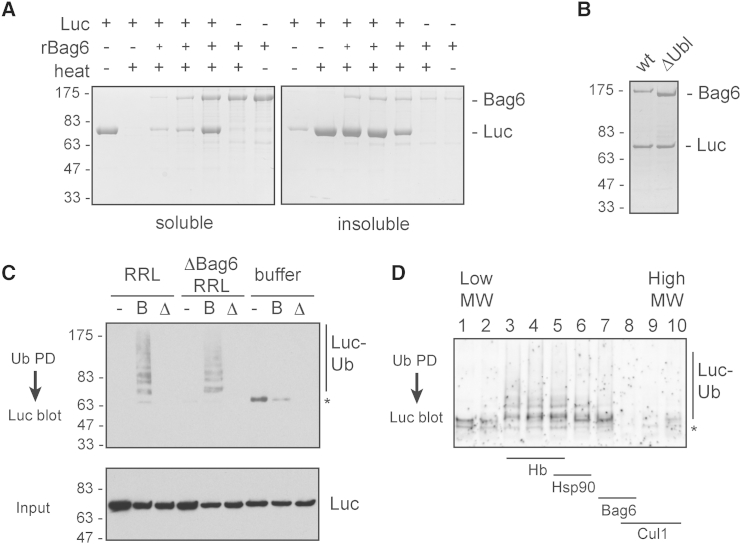
Purified Bag6-Client Complexes Support Ubiquitination by the MLP Ligase (A) Luciferase (Luc) and/or Bag6 was mixed as indicated and either left at 4°C or heated to 42°C for 20 min. After centrifugation, the soluble and insoluble fractions were analyzed by SDS-PAGE and Coomassie staining. (B) Coomassie-stained gel showing Bag6-Luc or ΔUbl-Bag6-Luc complexes. (C) Soluble luciferase (−), Bag6-Luc complex (B), or ΔUbl-Bag6-Luc complex (Δ) was added to reticulocyte lysate (RRL), Bag6-depleted lysate (ΔBag6-RRL), or buffer; supplemented with E1, E2, ATP, and Flag-ubiquitin; and incubated at 37°C for 30 min. The reaction was analyzed by anti-luciferase immunoblot either directly (Input) or after FLAG immunoprecipitation. The position of luciferase-ubiquitin conjugates is indicated. Asterisk denotes unmodified luciferase that nonspecifically binds the resin, particularly in the absence of lysate. (D) Bag6-depleted lysate was fractionated over a 5%–25% sucrose gradient, and each fraction was tested for ubiquitination activity using Bag6-Luc complex as the substrate as in (C). The migration positions of hemoglobin (60 kD native size), Hsp90, Bag6 complex, and Cullin1 are indicated. See also Figure S2.

To do this, we took advantage of the earlier observation that Bag6 can bind and maintain the solubility of Firefly luciferase (Luc) upon its heat-mediated misfolding (Wang et al., 2011). Although Luc is not an MLP per se, its sequence contains two unusually hydrophobic regions with TMD-like qualities (Figures S2A and S2B). These regions are likely the cause of its rapid precipitation upon unfolding, and high affinity for Bag6. Thus, heating of purified Luc to 42°C results in its quantitative precipitation, while heating in the presence of rBag6 results in a dose-dependent maintenance of Luc solubility (Figure 2A). After removal of aggregates, a stoichiometric Bag6-Luc complex is obtained (Figure 2B), which we used for downstream assays. As a control for nonspecific Luc ubiquitination, we also prepared complexes with ΔUbl-Bag6, a mutant Bag6 lacking its N-terminal Ubl domain (Figure 2B). This domain was shown in earlier experiments not to not be involved in substrate binding but to be necessary for client ubiquitination (Hessa et al., 2011).

To determine if Luc is a substrate for Bag6-mediated ubiquitination, we added E1, E2 (UbcH5), Flag-ubiquitin, ATP, and cytosol and assayed Luc ubiquitination via anti-Luc immunoblotting after Flag pull-downs. A ladder of ubiquitinated Luc was observed in a cytosol-dependent manner (Figure 2C). Depletion of Bag6 from the cytosol had no effect on its ubiquitination activity toward Luc-Bag6 complexes, illustrating that a ligase, and not Bag6 itself, was being assayed. Importantly, Luc in complex with ΔUbl-Bag6 did not support any detectable Luc ubiquitination (Figure 2C), despite the fact that the lysate presumably contains many different quality control ligases. Thus, a purified Bag6-Luc complex facilitates the selective analysis of ubiquitination in this pathway, a reaction that depends on the Ubl domain of Bag6 to presumably recruit a specific E3 ligase.

Using this assay, we found that Bag6-dependent ligase activity is detected not only in reticulocyte lysate, but also HEK293T cell lysate (Figure S2C). The activity in both lysates fractionated similarly by multiple criteria: it binds to anion exchange and phenyl-Sepharose, but not cation exchange (Figures S2D–S2F, and data not shown). Sucrose gradient sedimentation suggested that the ligase has a native size of between 30 and 120 kD (Figure 2D), excluding most HECT and SCF ligases. However, the activity appeared to be somewhat labile, and proved challenging to purify through multiple chromatographic steps. Nevertheless, fractionation of the activity as a relatively homogeneous product by multiple types of fractionation strongly suggested that reticulocyte and HEK293T cytosol contains one primary Bag6-dependent ligase, and revealed sufficient characteristics to narrow down potential candidates in subsequent experiments.

### RNF126 Is a Bag6-Associated E3 Ligase

**Figure 3:**
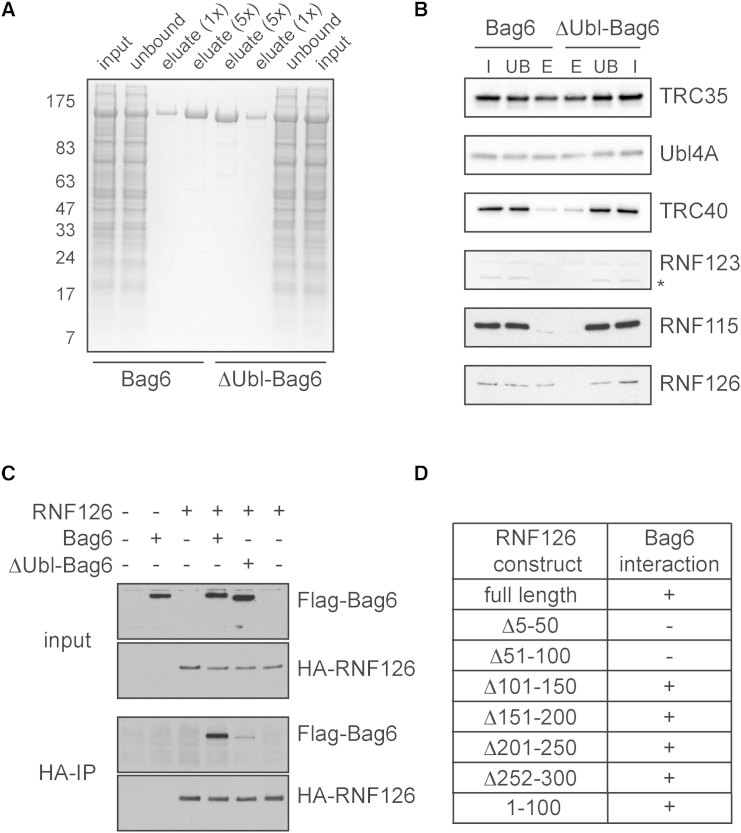
RNF126 Associates with Bag6 via Its Ubl Domain (A) Lysates from HEK293T cells transfected with either Flag-Bag6 or Flag-ΔUbl-Bag6 (input) were bound and eluted from Flag resin and the indicated fractions analyzed by SDS-PAGE and Coomassie staining. (B) The fractions from (A) were immunoblotted for the indicated proteins. Asterisk denotes nonspecific band. (C) The indicated proteins were coexpressed in HEK293T cells and analyzed by immunoblotting directly (input) or after IP with anti-HA antibodies (HA-IP). (D) Summary of interactions between Bag6 and various RNF126 constructs. Numbers refer to amino acid residues on RNF126. See also Figure S3.

While the majority of ligase activity could be readily separated from Bag6 (e.g., they migrate in different fractions of a sucrose gradient), a small amount of activity seemed to cofractionate with Bag6 in most experiments (data not shown). This suggested that the ligase interaction with Bag6 might be sufficiently long-lived to identify it via coassociation. We therefore overexpressed and affinity purified under native conditions FLAG-tagged Bag6 or ΔUbl-Bag6 (as a control) from HEK293T cells (Figure 3A). Although none of the copurified proteins were close to stoichiometric with overexpressed Bag6, immunoblotting did show recovery of known associating factors including TRC35, Ubl4A, and TRC40 (Figure 3B).

We therefore subjected the sample to MS/MS to identify all interacting E3 enzymes. Not surprisingly for a chaperone bait, a wide range of proteins were identified, including several ubiquitin ligases (Figure S3). Among these, some (e.g., RNF123) could be excluded because their association with Bag6 was not Ubl-domain specific (Figure 3B). Others (e.g., HUWE1 and Ubr family ligases) could be excluded based on their size, and yet others were not soluble cytosolic proteins (e.g., RMA1). The only ligases that remained after these exclusions were RNF126 and CHIP, an Hsc70-interacting ligase. Of these, we focused on RNF126, since CHIP was not detectable in reticulocyte lysate (data not shown), and was not depleted by phenyl-Sepharose (Figure S2E).

In addition to RNF126, two peptides from the highly related RNF115 were also detected, although this fell below the 95% confidence threshold. Nevertheless, both RNF126 and RNF115 are in the correct size range, widely expressed cytosolic proteins, found to arise in evolution coincident with Bag6, and verified by immunoblotting to be enriched in IPs of Bag6 relative to ΔUbl-Bag6 (Figure 3B). Analysis of the fractionation properties of RNF115 and RNF126 (Figures S2D–S2F) revealed that while both bind and elute from anion exchange, the highest ubiquitination activity is seen in the fraction containing the most RNF126 (and little or no RNF115). Furthermore, RNF115 does not bind phenyl-Sepharose, while RNF126 and MLP ubiquitination activity both bind and elute from this resin. Thus, RNF126 fractionation properties in HEK293T lysate (and reticulocyte lystate) generally match the Bag6-dependent ligase activity. Note that perfect concordance is not necessarily expected, since the different fractions have differing levels of deubiquitinase activities that partially confound precise quantification. Nevertheless, the similar fractionation properties of RNF126 and the Bag6-dependent ligase activity (Figures S2D and S2E), together with the Ubl domain-dependent RNF126 interaction with Bag6 (Figure 3B), made this a strong candidate for the MLP ligase.

Coimmunoprecipitation studies of epitope-tagged Bag6 and RNF126 expressed in cultured cells verified their specific interaction (Figure 3C). This interaction was markedly reduced (but not entirely eliminated) by deleting the Ubl domain of Bag6 (ΔUbl). RNF126 contains two recognizable domains: a Zn finger domain near the N terminus (residues 10–40), and a RING domain near the C terminus (residues 229–270) shown before to possess E3 ligase activity (Zhi et al., 2013). Deletion constructs of RNF126 in the Zn finger region abolished the Bag6 interaction, while a construct encoding only the first 100 residues of RNF126 (RNF126F) could be coimmunoprecipitated with Bag6 (Figure 3D). Thus, the N-terminal Ubl domain of Bag6 interacts with the N-terminal Zn finger-containing domain of RNF126. Further structural studies will be needed to analyze the Bag6-RNF126 interaction in molecular detail.

### Role for RNF126 in Bag6-Dependent Ubiquitination

**Figure 4:**
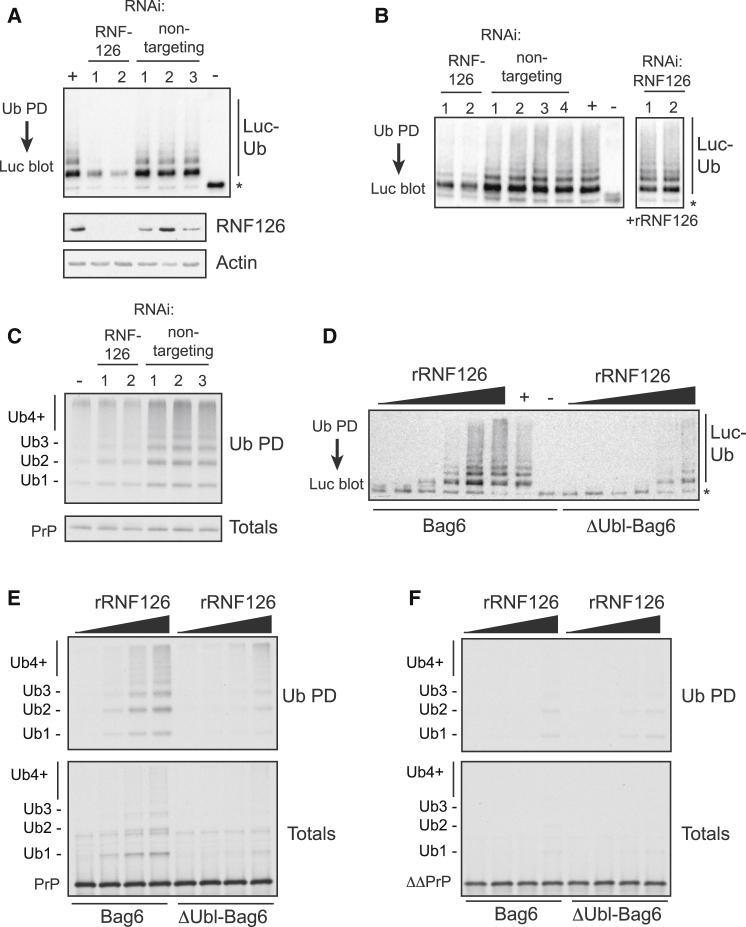
RNF126 Is Necessary and Sufficient for Bag6-Mediated Client Ubiquitination (A) Lysates prepared from HEK293T cells treated with the indicated siRNAs were used in ubiquitination assays of Bag6-Luc (top panel). The same lysates were immunoblotted for RNF126 (middle) or Actin (bottom). Untransfected cells were used as positive control for ubiquitination (+). The negative reaction (−) contains all components except lysate. (B) Analysis similar to in (A), with recombinant RNF126 (rRNF126, 0.1 μM final concentration) added prior to the assay to lysates 1 and 2 where indicated. (C) PrP was translated in phenyl-depleted lysate supplemented with recombinant Bag6, and this sample was used as the substrate in ubiquitination assays of HEK293T lysates as in (A). The reaction products were analyzed directly (Total) or after ubiquitin pull-downs (Ub-PD). (D) Various amounts of rRNF126 (final concentrations of 0.3, 1, 3.1, 9.8, 31, and 98 nM) were added to reactions containing E1, E2, ATP, Flag-ubiquitin, and either Bag6-Luc complex (Bag6) or ΔUbl-Bag6-Luc complex (ΔUbl-Bag6). Ubiquitinated products were immunoprecipitated via Flag resin and immunobloted for luciferase. As a positive control, RRL was used as the source of the ligase (+). (E) PrP was translated in phenyl-depleted lysate supplemented with recombinant Bag6 or ΔUbl-Bag6 in the presence of increasing amounts of rRNF126 (final concentrations of 0, 12, 39, and 118 nM). The translation products were analyzed directly (Total) or after ubiquitin pull-downs (Ub-PD). (F) Assay as in (E), but using ΔΔPrP as the substrate. See also Figure S4.

To examine the functional relevance of RNF126, we combined siRNA knockdowns with biochemical assays. Using Bag6-Luc as the substrate, we found that ubiquitination activity in HEK293T cytosol was reduced if the cells were first treated with either of two independent siRNAs against RNF126 (Figure 4A). Immunoblotting confirmed that RNF126 was knocked down by these siRNAs (Figure 4A). Neither of three nontargeting siRNAs had any effect. Adding back recombinant RNF126 (purified from *E. coli*; Figure S4A) to the siRNA-treated lysates restored their ubiquitination activity (Figure 4B). The role of RNF126 was also examined for a bona fide MLP as the substrate. In this experiment, we depleted reticulocyte lysate of its endogenous ligase by phenyl-Sepharose, then translated PrP in the presence of rBag6 to generate rBag6-PrP complexes. Subsequent incubation of this sample with HEK293T cytosol led to increased PrP ubiquitination, while cytosol from RNF126 knockdown cells showed minimal activity above that seen in the absence of added cytosol (Figure 4C). Thus, it appears that RNF126 is required for optimal ubiquitination of Bag6-associated substrates.

Recombinant RNF126 (rRNF126) was sufficient for ubiquitination of Luc on Bag6, but was far less effective against Luc bound to ΔUbl-Bag6 (Figure 4D). The residual activity against ΔUbl-Bag6 complexes is likely due to incomplete disruption of RNF126 association (Figure 3C). Recombinant RNF126 was also active toward Bag6-PrP complexes, and this ubiquitination was strongly dependent on the Ubl domain of Bag6 (Figure 4E). As a further specificity control, we analyzed ΔΔPrP, a version of PrP that lacks its signal peptide and GPI anchoring signal and does not interact with Bag6 (Hessa et al., 2011). RNF126 showed very poor activity toward ΔΔPrP, and what little ubiquitination that was observed was not affected by deleting the Ubl domain of Bag6 (Figure 4F). Finally, we verified that rRNF126 has activity toward Bag6-Sec61β complexes in a Ubl domain-dependent manner (Figure S4B). Of note, rRNF126 showed a very similar juxtahydrophobic lysine preference seen in native reticulocyte lysates, further arguing that the primary Bag6-dependent ligase in this system is RNF126 (Figure S4C). Taken together with the above siRNA experiments, we conclude that RNF126 plays a role in Bag6-mediated ubiquitination in our biochemical assays.

### Dominant-Negative Inhibition of the Bag6 Pathway In Vitro

**Figure 5:**
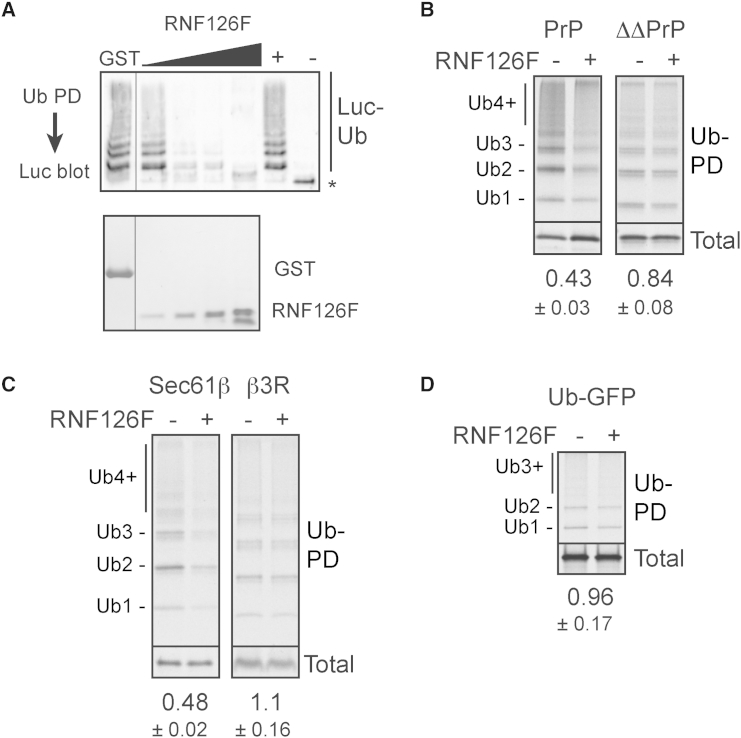
RNF126-Mediated Ubiquitination Is Specific to MLPs (A) The Bag6-Luc complex was incubated with E1, E2, ATP, Flag-ubiquitin, HEK293T lysate, and recombinant RNF126F at final concentrations of 2.2, 4.4, 8.8, and 22 μM. The products were analyzed after ubiquitin pull-downs by immunoblotting for Luc. Bottom panel shows Coomassie-stained gel of added proteins. GST was used as a control. (B) PrP or ΔΔPrP was translated in the presence or absence of 4.4 μM RNF126F. The products were visualized directly (bottom) after ubiquitin pull-downs (Ub-PD). Relative ubiquitination in the presence of RNF126F (mean ± SD, n = 3) is indicated below each panel. (C) Sec61β or Sec61β-3R was translated and analyzed as in (B). (D) Ub-GFP was translated and analyzed as in (B). See also Figure S5.

To understand the client specificity of RNF126 in cytosolic quality control, we turned to a dominant-negative inhibition strategy. We could show that the recombinant RNF126(1–100) fragment (RNF126F; Figure S5A) inhibited ubiquitination by RNF126 using either Bag6-Luc complex as a substrate (Figure 5A) or an MLP in the reticulocyte translation system (Figure S5B). The mechanism of inhibition appears to be via prevention of Bag6-RNF126 interaction as determined by coimmunoprecipitation analysis (Figure S5C). This dominant-negative strategy was used to probe substrate specificity of this pathway in the reticulocyte lysate system. As expected, we found that ubiquitination of Bag6-interacting proteins, defined by their hydrophobic elements, was selectively inhibited by RNF126F (Figures 5B–5D).

For example, ubiquitination of cytosolically mislocalized PrP was reduced to less than half by RNF126F, while almost no effect was seen for ΔΔPrP (Figure 5B). Similarly, RNF126F inhibited ubiquitination of cytosolic Sec61β, but not Sec61β(3R) in which the TMD is mutated to prevent Bag6 binding (Figure 5C). Importantly, Sec61β(3R)and ΔΔPrP were still perceived as misfolded by the lysate as evidenced by their ubiquitination. However, the pathway utilized was clearly different, since these clients continue to be ubiquitinated in a phenyl-depleted or Bag6-depleted lysate (data not shown), and are not inhibited appreciably by RNF126F (Figures 5B and 5C). Finally, a ubiquitin-GFP fusion protein (Ub-GFP) was also ubiquitinated in the system, but was not substantially inhibited by RNF126F (Figure 5D). Taken together, these findings argue that RNF126 is a quality control ligase that is selective to the Bag6 pathway for MLPs.

### RNF126 Knockdown Stabilizes a Bag6-Associated MLP in Cells

**Figure 6:**
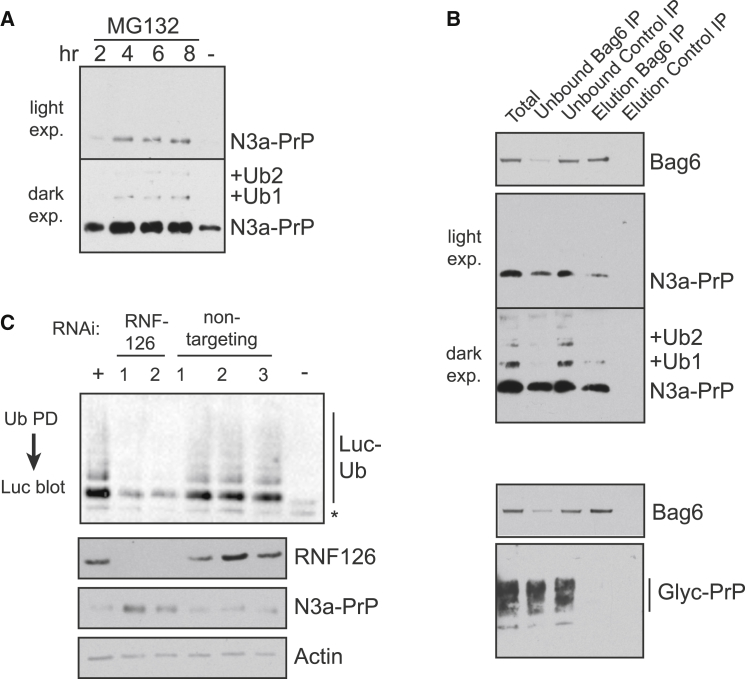
Stabilization of a Bag6-Associated MLP upon RNF126 Knockdown in Cells (A) HEK293T cells expressing N3a-PrP were incubated without or with 10 μM MG132 for the indicated times and analyzed by immunoblotting for N3a-PrP. Two exposures are shown and the ubiquitinated species indicated. (B) Lysates from cells expressing N3a-PrP (top panels) or wild-type PrP (bottom panels) were subjected to immunopurification using anti-Bag6 or control antibodies. Bag6, N3a-PrP, and PrP were followed during the purification by immunoblot. Two exposures of N3a-PrP are shown. (C) HEK293T cells expressing N3a-PrP were transfected with the indicated siRNAs for 72 hr. The lysates were analyzed for ubiquitination activity toward Bag6-Luc (top), and for RNF126, N3a-PrP, and actin by immunoblot (bottom). See also Figure S6.

To verify this conclusion in vivo, we generated an MLP in cultured cells and assessed its interaction with Bag6 and dependence on RNF126 for degradation. A signal sequence mutant (termed N3a) of mammalian PrP was previously shown to fail translocation in vitro (Kim et al., 2002), and was partially stabilized in cells upon Bag6 knockdown (Hessa et al., 2011). Expression of N3a-PrP was verified to be very low (detectable only with long exposure), but markedly increased when the proteasome was inhibited (Figure 6A). Immunopurification of Bag6 from N3a-PrP-expressing cells, even in the absence of proteasome inhibition, copurified N3a-PrP (Figure 6B). Glycosylated wild-type PrP was not found in the Bag6-immunopurified sample (Figure 6B), arguing against a postlysis interaction. Quantification of the Bag6 coIP experiment revealed that capture of ∼80% of cellular Bag6 recovered ∼50% of N3a-PrP, indicating that at least 60% of mislocalized PrP engages Bag6. Since RNF126 appears to be the primary Bag6-dependent ligase for mislocalized PrP (Figure 4C), these data suggest that most mislocalized PrP uses the Bag6-RNF126 system under normal conditions.

Knockdown of RNF126 (with either of two siRNAs) stabilized N3a-PrP (Figure 6C). Quantification via serial dilutions showed ∼3-fold increase in N3a-PrP levels with RNF126 knockdown, and an additional ∼3-fold increase with proteasome inhibition (Figure S6). Presumably, N3a-PrP (and other Bag6-associating clients) is degraded by other partially redundant cytosolic quality control pathway(s) in the absence of RNF126 and/or Bag6. This conclusion is consistent with the observation that in vitro, Bag6-dependent substrates are still ubiquitinated when Bag6 and/or RNF126 are depleted (e.g., in phenyl-depleted lysate). However, ubiquitination in the absence of the Bag6 pathway occurs more slowly and is less efficient, presumably because these alternate pathways are not optimized for MLPs. Future work will be needed to identify the full complement of cytosolic quality control pathways. Nevertheless, we can conclude that, in line with the in vitro studies, Bag6 clients rely on RNF126 for optimal ubiquitination and degradation in cultured cells. This is further supported by the finding that N3a-PrP ubiquitination is reduced in cells knocked down for RNF126 (Figure S6).

## Discussion

In this study, we have identified RNF126 as a ubiquitin ligase involved in cytosolic protein quality control. Based on the strategies used for its identification, validation in vitro using the recombinant protein, and its direct interaction with Bag6, we propose that RNF126 is relatively selective for the MLP pathway of quality control. Although we did not test an exhaustive set of misfolded proteins, RNF126 does not appear to target misfolded proteins lacking highly hydrophobic domains. Indeed, its appearance in evolution at the same time as Bag6 would suggest that the two may operate as a unit for dealing with MLPs in more complex metazoan organisms.

Our analysis indicates that RNF126 accesses MLPs via Bag6. While high concentrations of recombinant RNF126 can ubiquitinate MLPs in the absence of Bag6 (our unpublished data), we believe this to be nonspecific, since similar results were obtained with other ligases. Nevertheless, we cannot exclude the possibility that under some circumstances, RNF126 can directly interact with its targets for ubiquitination. This may be the case in instances where RNF126 appears to be used for regulated degradation (Delker et al., 2013; Zhi et al., 2013). Future work investigating the complete repertoire of RNF126 clients will be needed to draw general conclusions regarding its specificity.

It is curious that the ubiquitination of MLPs exhibits a degree of lysine specificity. The apparent favoring of lysines immediately adjacent to the Bag6 binding site may indicate that RNF126 positions the E2∼ubiquitin conjugate close to the Bag6 substrate binding domain. The juxtahydrophobic preference is noteworthy given that hydrophobic domains of MLPs are commonly flanked by lysines as part of the “positive-inside” rule (Wallin and von Heijne, 1998). Thus, the architecture of the Bag6-RNF126 complex may have evolved to exploit this feature of most clients for this pathway. Future work is needed to identify the Bag6 substrate binding domain and its relationship to the ubiquitination machinery.

The coupling of RNF126 to its clients via a chaperone adaptor is conceptually similar to CHIP, the other main metazoan quality control ligase characterized to date (Ballinger et al., 1999; McDonough and Patterson, 2003). CHIP interacts with chaperones of the Hsp70 family as well as Hsp90 to access many of its clients (Connell et al., 2001). The chaperone-CHIP complex can also interact with Bag1, Bag2, and Bag3 to further modulate its localization and/or activity (Kettern et al., 2010). Based on the observation that Bag6 prefers clients with long hydrophobic domains (Hessa et al., 2011; Mariappan et al., 2010) while the Hsp70/90 chaperones bind much shorter hydrophobic patches (Fourie et al., 1994; Jackson, 2013; Rüdiger et al., 1997), we believe that these two systems of quality control are largely nonoverlapping under normal circumstances.

Nevertheless, the chaperone-based system may deal with MLPs in the absence of a functioning Bag6 pathway. Indeed, depletion of Bag6 in the in vitro translation system does not lead to precipitation or aggregation of MLPs, but rather to their association with yet-unidentified binding partners (our unpublished data). At least some of these complexes seem to lead to MLP ubiquitination, albeit more slowly and less efficiently than the Bag6-RNF126 system. This would explain why depletion of either Bag6 or RNF126 in cells does not lead to complete stabilization of MLPs to the level of proteasome inhibition. It is likely that the cytosol contains several quality control pathways operating in parallel, each with their preferred client base (Buchberger et al., 2010; Fang and Mayor, 2012; Rodrigo-Brenni and Hegde, 2012). These pathways may be capable of substituting for other pathways in their absence or saturation, providing robustness to cellular quality control. Such a system of partially overlapping but distinct pathways also appears to exist in the endoplasmic reticulum, particularly in the more complex mammalian system (Araki and Nagata, 2011; Mehnert et al., 2010).

Beyond the RNF126 and CHIP pathways, other cytosolic quality control systems have been characterized primarily in yeast. Here, the ligases Ubr1, San1, and Hul5 appear to be the main cytosolic quality control ligases, while Ltn1 operates at stalled ribosomes (Bengtson and Joazeiro, 2010; Eisele and Wolf, 2008; Gardner et al., 2005; Heck et al., 2010). These pathways are relatively poorly understood at this time with respect to their client range, mechanism of client recognition, and potential interaction with chaperones. While the mammalian Ltn1 homolog Listerin has been shown to function similarly (Shao et al., 2013), the mammalian homologs of Ubr1, San1, and Hul5 remain poorly characterized with respect to quality control. Thus, a major area for future study will be to identify all of the major cytosolic quality control pathways in both model (e.g., yeast) and mammalian systems. Our identification of RNF126 and placement in the MLP pathway advances this goal.

In addition to a role in cytosolic quality control of MLPs, Bag6 has also been implicated in ER-associated degradation (ERAD) (Claessen and Ploegh, 2011; Wang et al., 2011). Here, Bag6 appears to bind and maintain the solubility of proteins dislocated from the ER membrane. Since dislocation occurs after ubiquitination, it may be that the main function of Bag6 in ERAD is to prevent client aggregation en route to the proteasome. Alternatively, dislocation may be accompanied by deubiquitination in order to pass through the p97/Cdc48 pore (Ernst et al., 2009). In this instance, Bag6 would be bound to a nonubiquitinated dislocated client that may need reubiquitination for proteasome targeting. If this were the case, there may be a role for RNF126 late in the ERAD pathway. This possibility merits attention in future studies.

Why are the Bag6-RNF126 and chaperone-CHIP pathways only in metazoans? One apparently distinctive feature of both pathways is their direct link to biosynthetic factors. The Bag6 complex is part of the TA protein insertion pathway (Mariappan et al., 2010), while Hsp70 and Hsp90 are involved in cytosolic protein folding (Kim et al., 2013). One might speculate that embedding ubiquitin ligases within biosynthetic pathways seamlessly targets failed maturation products for degradation (Rodrigo-Brenni and Hegde, 2012). This may minimize the opportunity for off-pathway interactions or aggregation, a particularly detrimental outcome for long-lived organisms with many slowly dividing and postmitotic cells. Yeast, with their highly robust Hsp104 disaggregase (Doyle and Wickner, 2009) and ability to avoid aggregate inheritance during cell division (Zhou et al., 2011), may not require direct coupling of biosynthesis and quality control. Metazoan quality control systems may typically operate as a highly coordinated triage reaction carried out by dynamic complexes with seemingly competing factors such as chaperones and ligases (Rodrigo-Brenni and Hegde, 2012).

Our identification of RNF126 as part of the Bag6 complex now paves the way for understanding the mechanistic basis of one such triage system. Nascent TA proteins engage this complex and can either be transferred to the targeting factor TRC40 (Mariappan et al., 2010) or ubiquitinated by RNF126. The latter reaction is favored when TRC40 is absent, while excess TRC40 minimizes ubiquitination (Hessa et al., 2011). The outcome may be further influenced by yet other Bag6-interacting factors such as the cochaperone SGTA (Leznicki and High, 2012). How this triage “decision” is made will likely serve as a paradigm for comparable events in other quality control systems, and represents an important topic for future study.

## Experimental Procedures

### Plasmid and Antibodies

SP64 plasmids encoding TR-β, Sec61β, Sec61β-3R, PrP, ΔΔPrP, and Ub-GFP were described (Ashok and Hegde, 2008; Dantuma et al., 2000; Mariappan et al., 2010; Stefanovic and Hegde, 2007). Sec61β and TR-β lysine mutants were made by mutagenesis. Bag6-Flag, ΔUbl-Bag6-Flag, human PrP, and hamster N3a-PrP were described (Hessa et al., 2011; Kim et al., 2002). Bag6 cDNA was cloned with an N-terminal His6 tag encoded in the oligos into the pFastBac dual vector (Invitrogen). RNF126 cDNA was cloned into pCDNA3.1 with a N-terminal 3xHA tag for expression in mammalian cells and into pET28 vector for expression in *E. coli*. RNF126 deletions and truncations were made by mutagenesis. Depletion of RNF126 in HEK293T cells was achieved by siRNAs from Invitrogen: #1, CAUCCCGGACGGUACUUCUGCCACU; #2, CAGCAGCUCGUCAACGGCAUCAUCA. Commercial antibodies are as follows: luciferase (Abcam), RNF123 (Abcam), RNF115 (Abcam), Flag M2 (Sigma-Aldrich), actin (Sigma-Aldrich), and CHIP (Bethyl). Antibodies against Bag6, TRC35, Ubl4A, TRC40, HA, PrP (3F4), and hamster PrP (13A5) have been described (Hegde et al., 1998; Mariappan et al., 2010). Anti-RNF126 antibodies were raised (by Eurogentec) against a C-terminal peptide (TGQNTATNPPGLTGVS) conjugated to KLH and affinity purified using the peptide. Anti-Flag and anti-HA affinity resins were from Sigma-Aldrich.

### In Vitro Translation

In vitro translation in rabbit reticulocyte lysate (RRL) was as previously described (Sharma et al., 2010). Phenyl-depleted lysate (ph-RRL) was generated by passing 700 μl RRL over 400 μl phenyl-Sepharose resin by gravity at 4°C and collecting the peak flowthrough fractions. ΔBag6-RRL was generated by incubating 800 μl RRL with 200 μl anti-Bag6 resin. Translations were for 1 hr at 32°C unless stated otherwise. Reactions were supplemented with rBag6, rΔUbl-Bag6, or rBag6 complex to similar levels as RRL (determined via semiquantitative immunoblot). For direct analysis or ubiquitin pull-downs, the reactions were stopped by addition of 1% SDS, boiled, and diluted 10-fold in IP buffer: 0.5% Triton X-100, 50 mM HEPES (pH 7.4), 100 mM NaCl, and 10 mM imidazole. For native immunoprecipitations the proteins were placed on ice, and all other manipulations were carried out at 0°C–4°C.

### Recombinant Proteins

His-tagged Bag6 or ΔUbl-Bag6 was expressed in Sf9 cells using baculovirus and purified using metal-affinity chromatography on Co^2+^ immobilized on chelating Sepharose (Amersham). Preparation of recombinant Bag6 complex will be described in detail elsewhere, but involved production of Ubl4A and TRC35 in *E. coli*, followed by their assembly with Bag6 produced in mammalian cells. His-tagged RNF126 and RNF126F were expressed in BL21 (DE3) cells in the presence of 250 μM ZnCl_2_ and purified using metal-affinity chromatography on cobalt resin. Purified Bag6-luciferase complex was made by mixing equimolar amounts of rBag6 (or ΔUbl-rBag6) and luciferase, heating to 42°C for 20 min, and removing aggregates by centrifugation (10 min, 20,000 × g). Other proteins (GST-Ube1, UbcH5c, His-ubiquitin, and Flag-ubiquitin) were purchased from Boston Biochem. Luciferase was from Promega.

### Ubiquitination Reactions

Ubiquitination during translation used 10 μM His-ubiquitin. Posttranslational ubiquitination reactions were supplemented with a ubiquitination mix consisting of 100 nM GST-Ube1, 250–375 nM UbcH5a, 10 μM His-ubiquitin, and an ATP-regenerating system (1 mM ATP, 10 mM creatine-phosphatase, 40 μg per ml creatine-kinase in buffer). Reactions were incubated at 32°C for 1 hr, stopped by the addition of 1% SDS, and boiled. Purified Bag6-Luc complex ubiquitination was done by preincubating the ubiquitination mix (substituting Flag-ubiquitin for His-ubiquitin) for 15 min prior to addition of ligase source (lysate, fractionated lysate, or rRNF126) and substrate. Reactions were for 1 hr at 37°C. Reactions were stopped with 1% SDS, boiled, and diluted 10-fold in IP buffer: 1% Triton X-100, 50 mM HEPES (pH 7.4), and 100 mM NaCl. Lysates from siRNA experiments were assayed for 10 min. Reactions with rRNF126 were for 20 min.

### Miscellaneous Biochemistry

For fractionation (Figure S2), HEK293T cells were swelled for 15 min on ice in hypotonic buffer (10 mM HEPES [pH 7.4], 10 mM KAc, 1 mM MgAc_2_, PMSF, DTT, and protease inhibitor cocktail from Roche), lysed by douncing, and clarified by centrifugation at 600 × g for 10 min at 4°C. The lysate was adjusted to 50 mM HEPES (pH 7.4), 100 mM KAc, and 2 mM MgAc_2_ prior to further fractionation. Bag6-Flag or ΔUbl-Bag6-Flag was overexpressed after transient transfection of HEK293T cells, a lysate prepared as above, captured with anti-Flag resin, and eluted with 0.2 mg/ml FLAG peptide. Gel slices were analyzed by trypsin digestion and mass spectrometry.

### Cell Culture

HEK293T cells were grown in DMEM plus with 10% FBS. Transfections utilized TransIT293 (Mirus). siRNA transfections utilized Lipofectamine RNAiMAX (Invitrogen). PrP and/or N3a-PrP was overexpressed after transient transfection of HEK293T cells. After 48 hr the cells were transfected with siRNAs against RNF126 or nontargeting sequences. Seventy-two hours later the cells were split evenly in two. One set was lysed with 1% SDS and boiled to analyze total proteins. The other was lysed with lysis buffer (0.5% Triton X-100, 50 mM HEPES [pH 7.4], 100 mM NaCl, 2 mM MgCl2, PMSF, DTT, protease inhibitor cocktail from Roche) to assay ligase activity.

## Author Contributions

M.C.R.-B. and R.S.H. designed the project. M.C.R.-B. carried out all the experiments except the Bag6-RNF126 interaction experiment (E.G.) and Bag6-PrP interaction (R.S.H.). R.S.H. and M.C.R.-B. wrote the paper.

## Supplemental Information

Document S1. Six Figures

Document S2. Article plus Supplemental Information
