# Supplementary material for: Cytosolic Quality Control of Mislocalized Proteins Requires RNF126 Recruitment to Bag6
Source: Mol Cell. 2014 Jul 17;55(2):227–37. doi: 10.1016/j.molcel.2014.05.025 (PMC4104027; doi:10.1016/j.molcel.2014.05.025)
Supplement: Document S1. Six Figures [file mmc1.pdf]

Molecular Cell, Volume *55*

Supplemental Information

Cytosolic Quality Control of Mislocalized Proteins Requires RNF126 Recruitment to Bag6

Monica C. Rodrigo-Brenni, Erik Gutierrez, and Ramanujan S. Hegde

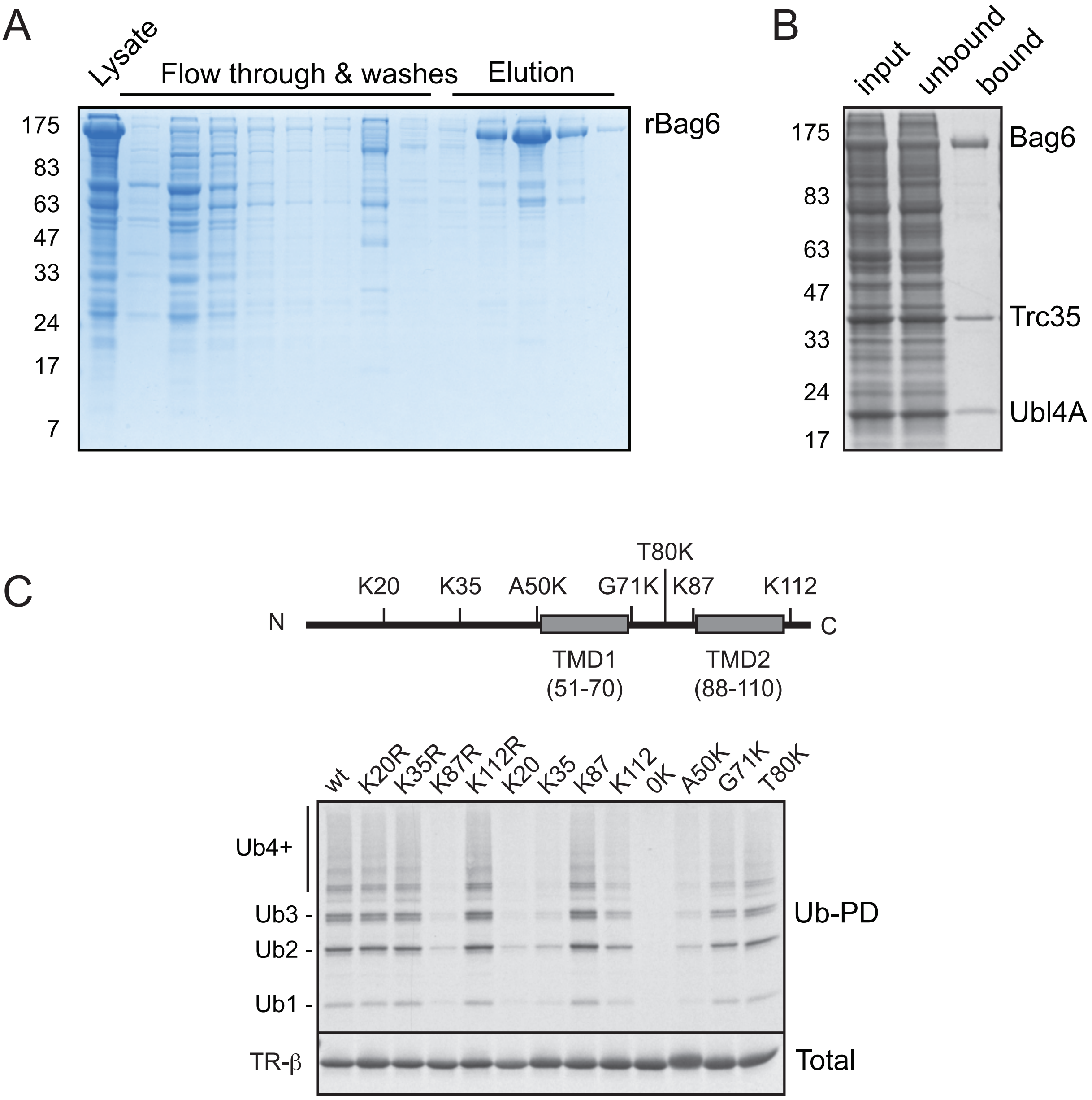

Figure S1 (related to Figure 1) - Characterization of a Bag6-dependent ubiquitin ligase for MLPs. (A) Recombinant His-tagged Bag6 was expressed in insect cells and purified under native conditions over Co+2-charged chelating sepharose resin. After binding, the resin was extensively washed with up to 25mM imidazole (Flow through and washes). Bound material was eluted with 200mM imidazole (Elutions). (B) HEK293T cells were transfected with FLAG-tagged Bag6 and the resulting lysate was incubated with excess purified TRC35 and Ubl4A (both purified from E. coli) to permit ternary complex formation. The complex was purified via anti-FLAG affinity resin and eluted using competing peptide. (C) Diagram depicting location of lysines and transmembrane domains (TMD) in TR-β (top). TR-β, lysine-to-arginine mutants (KxR, where x denotes residue number), or single lysine constructs (Kx, where x denotes residue number of single lysine present) were in vitro translated and analyzed after Ub-PD. The bottom panel shows total translation products.

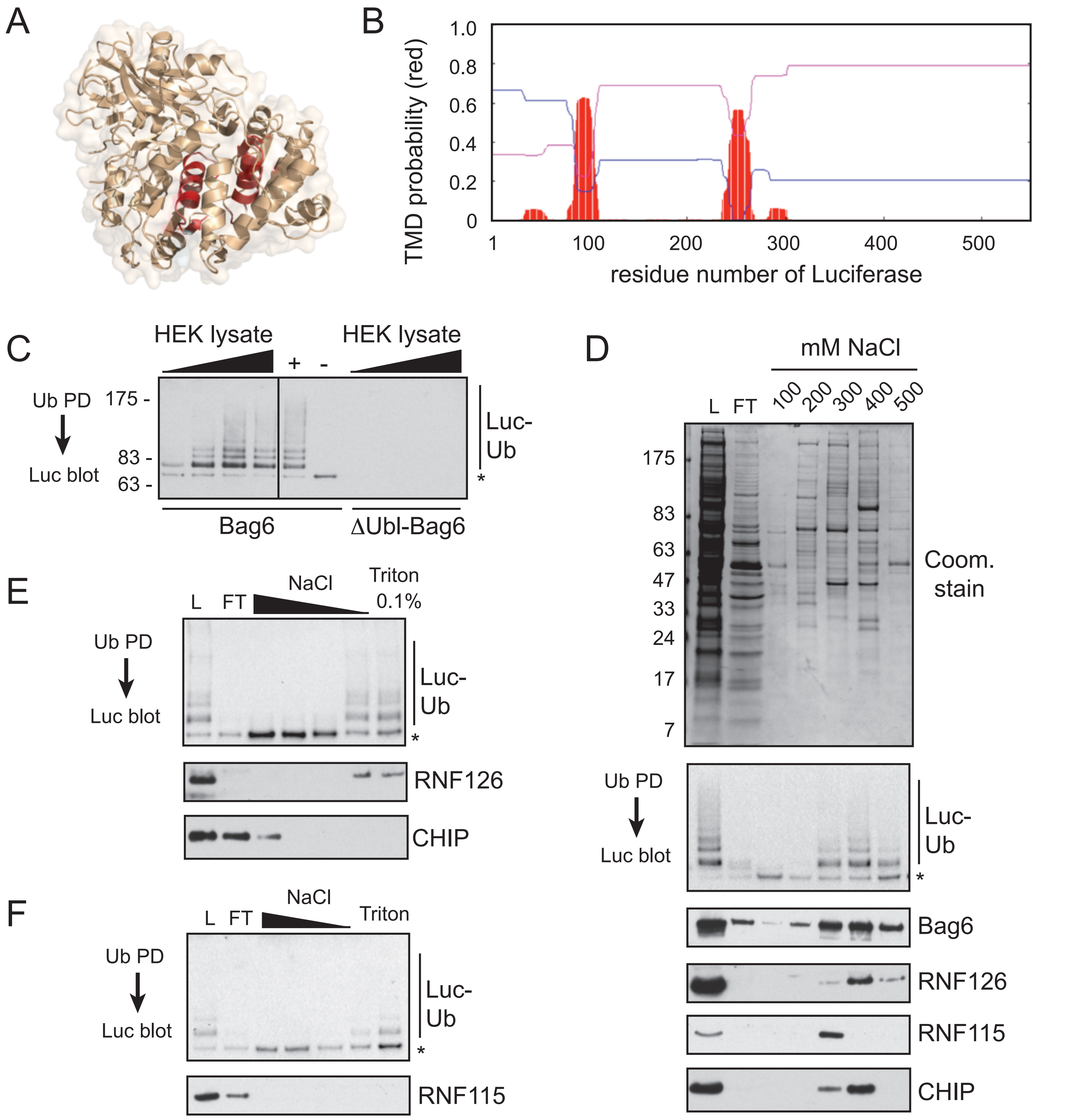

Figure S2 (related to Figure 2) - Characterization of the MLP ligase using Luc-Bag6 complex as a client. (A) Depiction of Luciferase structure (PDB 1LCI) with the two most hydrophobic helices indicated in red. Note that these are fully buried in the native structure. (B) Analysis of Luciferase sequence for transmembrane domains using the TMHMM algorithm. Red denotes transmembrane domain (TMD) probability. Note that the two most hydrophobic domains (indicated in panel A) show high transmembrane domain-like tendency. (C) Luc-Bag6 or Luc-ΔUbl-Bag6 complexes prepared as in Figure 2B were incubated in the presence of E1, E2, ATP and Flag-ubiquitin with increasing amounts of HEK293T lysate. Ubiquitinated products were purified via Flag resin and immunoblotted for luciferase. RRL was used as the positive control (+), and no lysate was added to the negative control (-). Asterisk denotes unmodified luciferase bound non-specifically to the resin. (D) Lysate from HEK293T cells was loaded onto Q resin and eluted by increasing the salt concentration in steps (100-500mM salt). L is the lysate and FT is the flow-through. Each fraction was analyzed by coomassie staining, by ubiquitination assays of Luc-Bag6 complexes, and by immunoblotting for Bag6, RNF126, RNF115 and CHIP. (E) The 400mM elution from panel D was fractionated by phenyl sepharose. The flow through fraction was collected and the resin eluted by progressively lowering the salt, followed by elution with Triton X-100. L is the load fraction and FT is flow-through. The fractions were analyzed for their ability to support ubiquitination of Luc-Bag6 complexes, and by immunoblotting for RNF126 and CHIP. Note that the ubiquitination activity and RNF126 both bind phenyl sepharose and only elute with Triton-X 100. By contrast, CHIP does not bind under these conditions. (F) The 300mM elution fraction from panel D was fractionated by phenyl sepharose as in panel E. The fractions were analyzed for their ability to support ubiquitination of Luc-Bag6 complexes, and by immunoblotting for RNF115. Note that the ubiquitination activity quantitatively binds phenyl sepharose, while RNF115 does not.

Protein name	MW (kDa)	Weighted spectral counts Bag6 sample	% coverage Bag6 sample	Weighted spectral counts ΔBag6 sample	% coverage ΔBag6 Sample
HUWE1	482	6.93	1.60	1.94	0.78
Ubr5	309	4.37	1.80	2.82	0.79
Ubr3	212	22.74	6.3	21.95	9.2
Ubr2	201	7.49	3.3	9.71	5.1
RNF123	149	4.60	1.2	23.12	4.0
WWP2	99	1.07	2.9	1.96	4.3
HECTD3	97	1.70	3.3	1.96	4.8
RNF12	69	0	N.A.	1.63	3.7
TRAFD1	65	1.20	8.9	0.72	2.1
TRIM27	58	4.46	4.9	8.19	9.6
TRIM21	54	5.18	8.8	7.21	11.0
TRIM11	53	0	N.A.	2.94	6.2
FANCL	43	2.87	8.5	3.90	8.5
RNF126	36	9.28	12	3.53	19
CHIP	35	13.32	15	5.71	22
CCNB1Ip1	32	0	N.A.	2.34	6.9
RNF5 (RMA1)	20	6.68	18	3.00	8.3

Figure S3 (related to Figure 3) – Mass spectrometry analysis of Bag6-interacting ubiquitin ligases. List of all ubiquitin ligases identified by mass spectrometry to >95% confidence in the affinity purified samples of Bag6 and ΔUbl-Bag6 from Figure 3A. The ligases are sorted by molecular weight from largest to smallest.

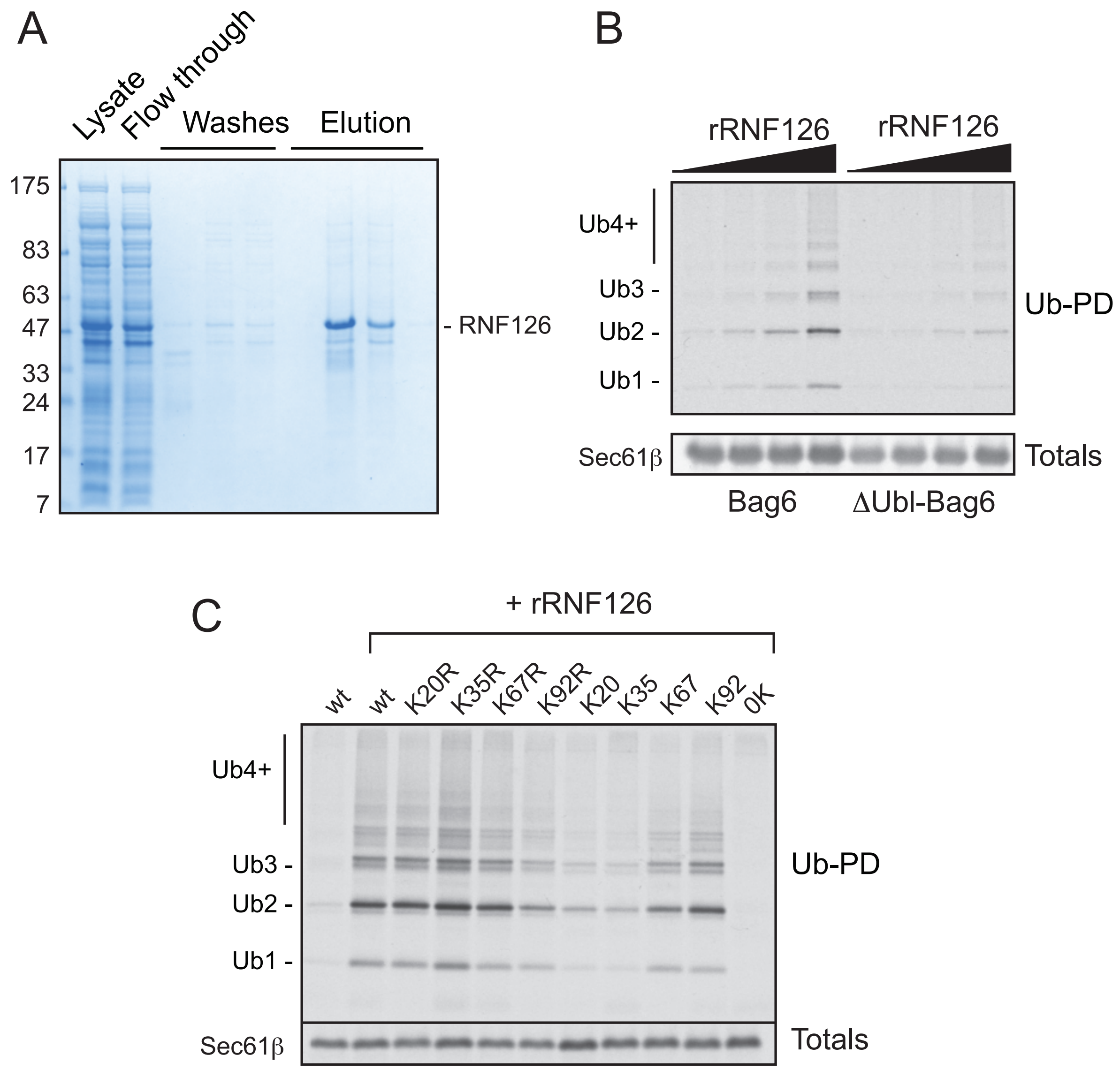

Figure S4 (related to Figure 4) - Characterization of purified RNF126. (A) Coomassie-stained gel of His-tagged RNF126 purification from E. coli. (B) Sec61β was translated in phenyl-depleted lysate supplemented with recombinant Bag6 or ΔUbl-Bag6. The samples were then subjected to ubiquitination assays with increasing concentrations of rRNF126 (0, 12, 39, and 118 nM). The translation products were analyzed after ubiquitin pulldowns (Ub-PD) followed by SDS-PAGE and autoradiography. The bottom panel shows total translation products. (C) Various lysine mutants in Sec61β (see Figure 1F) were translated in phenyl-depleted lysate supplemented with recombinant Bag6 and analyzed for their ability to be ubiquitinated by 39 nM rRNF126 as in panel B.

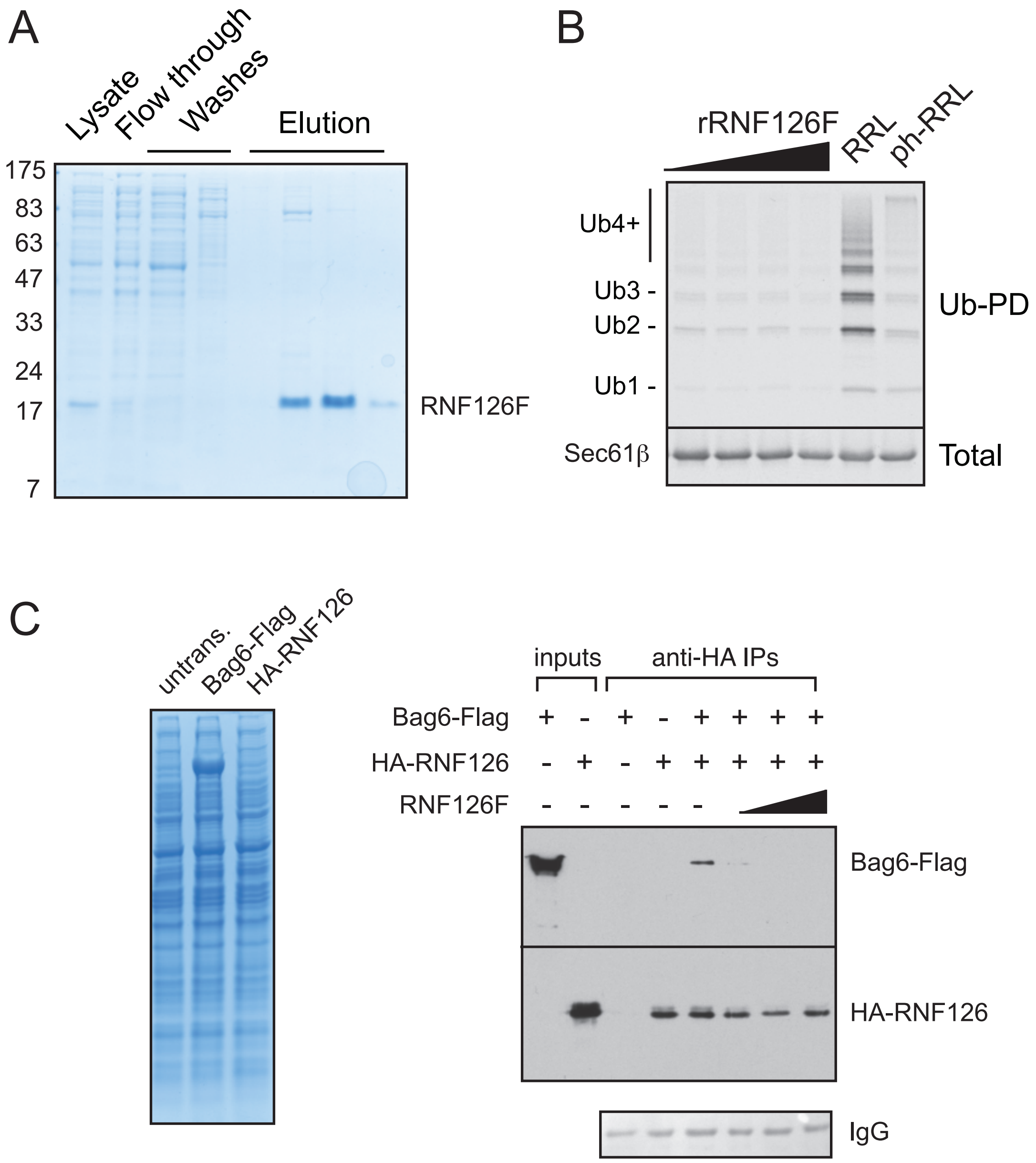

Figure S5 (related to Figure 5) - Characterizatin of RNF126F as a dominant-negative inhibitor. (A) Coomassie-stained gel of His-tagged $RNF126_{1\text{-}100}$ (RNF126F) purification from E. coli. (B) Sec61β was translated in the presence of increasing amounts of RNF126F (2.2, 4.4, 8.8, and 15.4 μM final concentration). The translation products were analyzed after ubiquitin pulldowns (Ub-PD) followed by SDS-PAGE and autoradiography. The bottom panel shows total translation products. For comparison translations in complete lysate (RRL) or phenyl-depleted lysate (ph-RRL) are included. (C) Lysates were prepared from cells expressing either FLAG-tagged Bag6 or HA-tagged RNF126 (left panel). These lysates (input) were mixed without or with different amounts of RNF126F (at 4.4, 8.8, and 17.6 μM), and subjected to immunoprecipitation with anti-HA antibodies (right panel). The input samples and IPs were separated by SDS-PAGE and immunoblotted with anti-HA and anti-FLAG to detect RNF126 and Bag6, respectively. Ponceau staining of the blot verified equal recovery of IgG in the IPs (bottom panel).

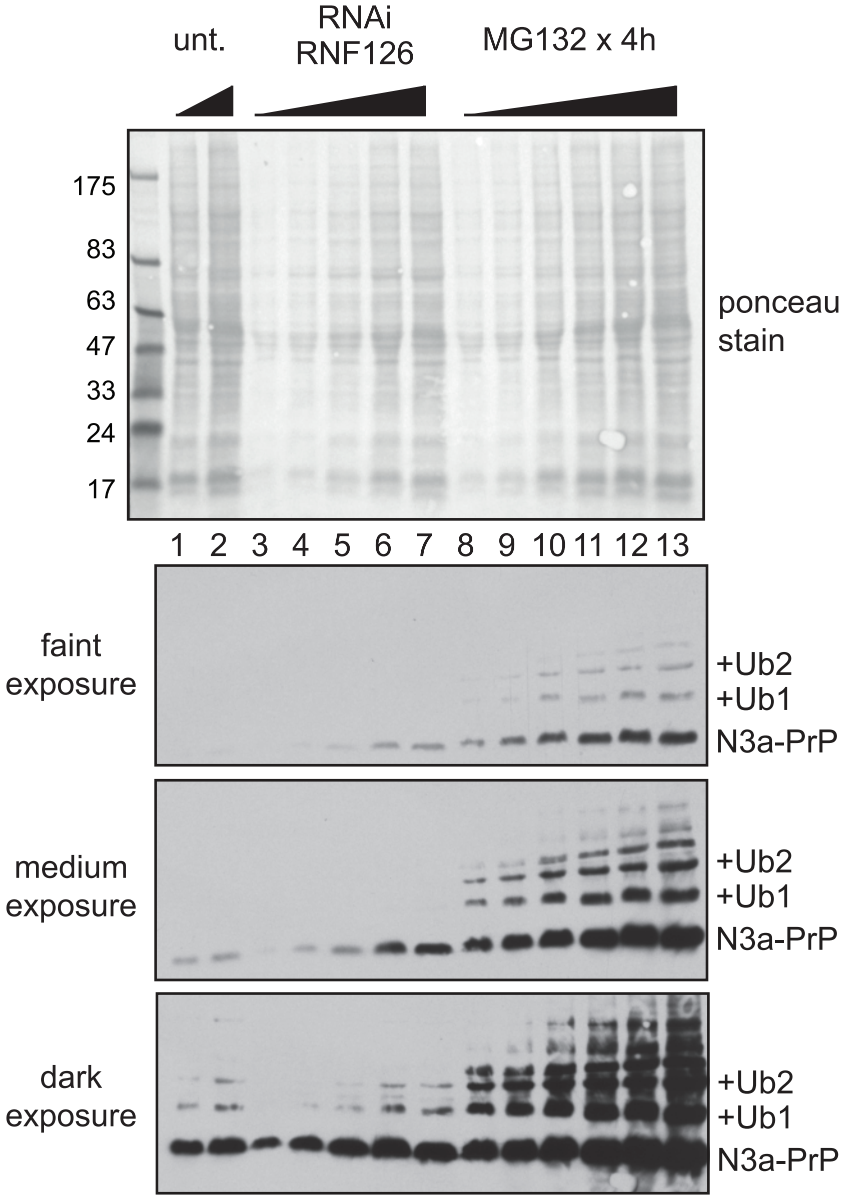

Figure S6 (related to Figure 6) - Quantification of N3a-PrP stabilization and ubiquitination. Total cell lysates were prepared from N3a-PrP expressing cells that were untreated, knocked down for RNF126, or treated with 10 μM MG132 for 4 hours. Serial dilutions of each lysate were analyzed by SDS-PAGE, transferred to nitrocellulose, and stained for total protein (top panel) before immunoblotting for N3a-PrP (bottom panels). Three exposures are shown. Unmodified N3a-PrP, as well as ubiquitinated species, are indicated. The relative amount of N3a-PrP for each treatment condition was quantified by determining the relative amounts of lysates needed to achieve an equal N3a-PrP signal by blotting. For example, the medium exposure shows that lanes 1 and 2 (untreated) give a very similar N3a-PrP signal to lanes 4 and 5 (RNF126 knockdown). Since lanes 1 and 2 have 2.94-fold and 2.90-fold more protein than lanes 4 and 5, respectively, we can estimate that RNF126 knockdown results in a 2.9-fold increase in N3a-PrP levels. Using similar calculations, we further conclude that N3a-PrP upon proteasome inhibition is ~3.1-fold higher than in the RNF126 knockdown. Inspection of the ubiquitinated species in the long exposure reveals that in samples that contain equal amounts of N3a-PrP (e.g., lanes 1 and 2 versus lanes 4 and 5), ubiquitination is lower in the RNF126 knockdown. The same conclusion is reached by appreciating that lanes containing equal amounts of ubiquitinated species (e.g., lane 2 versus 6) have a greater amount of unmodified substrate for the RNF126 sample. Thus, N3a-PrP ubiqutination is lower in RNF126 knockdown cells. It is also easily appreciated that proteasome inhibition results in a greater proportion of ubiquitinated species per substrate than RNF126 knockdown.
